# (23*R*,23^1^ *S*,25*S*)-23^1^,26-Ep­oxy-23-ethyl­furost-20(22)-en-3β-ol

**DOI:** 10.1107/S2414314623003449

**Published:** 2023-04-21

**Authors:** Gabriel Guerrero-Luna, María-Guadalupe Hernández-Linares, Maura Cárdenas García, Sylvain Bernès

**Affiliations:** a Instituto de Ciencias, Benemérita Universidad Autónoma de Puebla, Av. San Claudio y 18 Sur, 72570 Puebla, Pue., Mexico; b Laboratorio de Investigación del Jardín Botánico, Benemérita Universidad Autónoma de Puebla, Av. San Claudio y 18 Sur, 72570 Puebla, Pue., Mexico; c Facultad de Medicina, Benemérita Universidad Autónoma de Puebla, 13 Sur 2702, 72420 Puebla, Pue., Mexico; d Instituto de Física, Benemérita Universidad Autónoma de Puebla, Av. San Claudio y 18 Sur, 72570 Puebla, Pue., Mexico; Goethe-Universität Frankfurt, Germany

**Keywords:** crystal structure, tetra­hydro­pyran, sarsasapogenin, rearrangement reaction, hydrogen bond

## Abstract

The title compound, C_29_H_46_O_3_, is a steroid synthesized through a rearrangement of a sarsasapogenin derivative in acidic medium. The newly formed ring *F* is a tetra­hydro-2*H*-pyran heterocycle substituted by two methyl groups placed in equatorial positions. This ring displays a chair conformation, while di­hydro­furan ring *E*, to which it is bonded, has an envelope conformation. The mol­ecules are associated by weak O—H⋯O hydrogen bonds to form chains running in the [101] direction in the crystal.

## Abstract 2

The title steroid includes a tetra­hydro-2*H*-pyran heterocycle bonded to the steroidal nucleus, resulting from a spiro­stan rearrangement.

## Graphical abstract

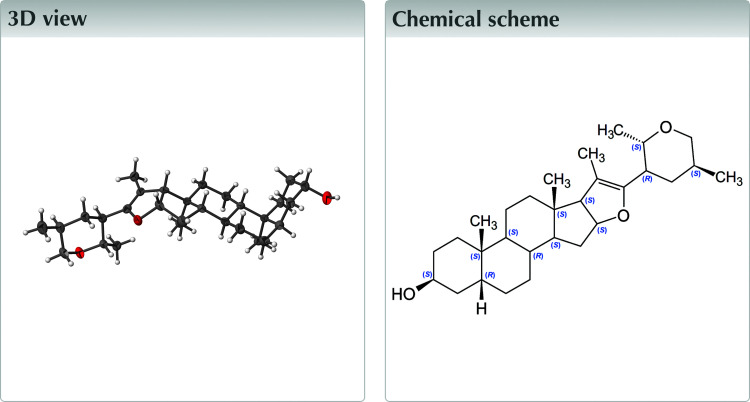

## Structure description

**Figure 1:**
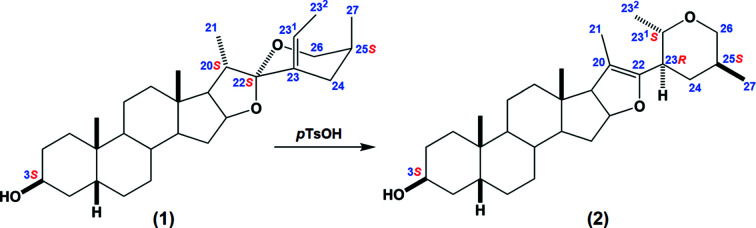
The synthesis of the title compound, **2**, starting from **1**. The atom-numbering scheme follows the recommendations of IUPAC (Moss, 1989 ▸). Key *R*/*S* configurations are displayed in red. *p*TsOH is *p*-toluene­sulfonic acid.

**Figure 2:**
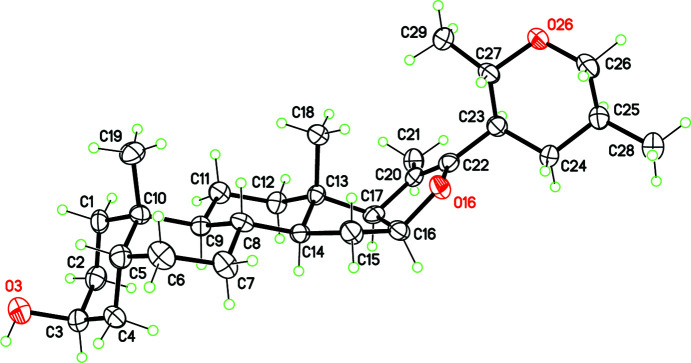
The mol­ecular structure of the title compound, **2**, with displacement ellipsoids for non-H atoms drawn at the 50% probability level.

The title steroid (**2**) was synthesized, starting from a derivative of sarsasapogenin (**1**), through a cleavage of ring *F* in acidic medium, followed by a Michael-type nucleophilic attack that affords a tetra­hydro-2*H*-pyran ring bonded to the steroidal *E* ring (Fig. 1 ▸). The crystal structure of **1**·0.5H_2_O has been reported previously (Viñas-Bravo *et al.*, 2003 ▸). On the other hand, the mechanism for the rearrangement **1**→**2** was described previously using a diosgenin derivative as substrate, instead of sarsasapogenin (del Río *et al.*, 2006 ▸). As expected from this mechanism, both methyl groups substituting the pyran ring in **2** are placed in equatorial positions, defining the stereochemistry for atoms C25 and C27 as *S*,*S* (Fig. 2 ▸). The pyran ring adopts a chair conformation, characterized by a puckering amplitude *q* = 0.578 (4) Å. Surprisingly, the Cambridge Structural Database (Version 5.43, with all updates; Groom *et al.*, 2016 ▸) does not contain any structure including the same heterocycle. However, many polysubstituted monocyclic tetra­hydro-2*H*-pyran structures have been characterized by X-ray diffraction, showing that the chair conformation is almost always stabilized (*e.g.* Burton *et al.*, 2007 ▸). Only a few exceptions to this rule are known, for some large mol­ecules with steric hindrance issues (*e.g.* Aydillo *et al.*, 2013 ▸).

The 2,3-di­hydro­furan ring *E* in **2** is close to being planar due to the presence of the C20=C22 double bond [1.324 (5) Å], also evidenced by a vibration at 1627 cm^−1^ in the IR spectrum. The conformation can be described as an envelope with atom C16 as the flap, which belongs to the C—C bond fusing the *D* and *E* rings. The *E* ring has a small puckering amplitude, *q* = 0.184 (4) Å. A very similar conformation was observed in other steroids having the same *E* ring (Shen *et al.*, 2013 ▸; Jeong & Fuchs, 1994 ▸). The remainder of the mol­ecular structure, *i.e.* the *A*/*B*/*C*/*D* steroidal nucleus, is identical to that of sarsasapogenin, with *cis*-fused *A*/*B* rings.

**Figure 3:**
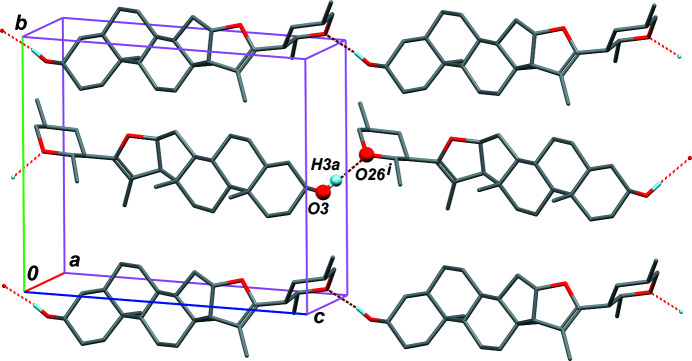
Part of the crystal structure of the title compound, **2**, showing chains formed *via* O—H⋯O hydrogen bonds (dashed bonds). For symmetry code (i), see Table 1 ▸.

The crystal structure is very simple, since it is based on a single weak O—H⋯O hydrogen bond, involving the hy­droxy group at C3 and the O-atom acceptor of the tetra­hydro-2*H*-pyran ring, O26 (Table 1 ▸ and Fig. 3 ▸). The mol­ecules form infinite chains, running in the [101] direction. Neighbouring chains in the crystal are related by the twofold screw axis parallel to [010] in the space group *P*2_1_.

The mol­ecular structure of **2** is embedded in a broader project aimed at targeting steroidal compounds which could inter­act with signalling pathways that control skeletal muscle atrophy and hypertrophy (Cohen *et al.*, 2015 ▸). Indeed, the web tool *SwissTargetPrediction* (Daina *et al.*, 2019 ▸) predicts that compound **2** presents a binding affinity for androgen and estrogen receptors, as well as for PI3K enzyme.

## Synthesis and crystallization

In a round-bottomed flask was dissolved 275 mg (0.62 mmol) of **1** and 150 mg of *p*TsOH (*ca* 0.9 mmol) in 5 ml of benzene, and this mixture was refluxed for 30 min. The crude was then evaporated and the resulting solid dissolved in CH_2_Cl_2_, washed with distilled H_2_O, dried over Na_2_SO_4_ and evaporated *in vacuo* to dryness. The residue was purified by chromatography over silica gel with hexa­ne/EtOAc (4:1 *v*:*v*) as eluent, to give 268 mg of **2** (97% yield). IR (ν, cm^−1^): 2998 (C—H), 1627 (C=C). ^1^H NMR (500 MHz, CDCl_3_): δ (ppm) 4.70 (1*H*, *m*, H-16), 4.08 (1*H*, *m*, H-3), 3.79 (1*H*, *ddd*, *J*_26eq,26ax_ = 11.0, *J*_26eq,25_ = 3.4 Hz, *J*_26eq,24eq_ = 1.4, H-26eq), 3.36 (1*H*, *dq*, *J*_23 (1),23_ = 9.54, *J*_23 (1),23 (2)_ = 6.2 Hz, H-23^1^), 2.98 (1*H*, *dd*, *J*_26ax,26eq_ = *J*_26ax,25_ = 11.0 Hz, H-26ax), 2.46 (1*H*, *d*, *J*_17,16_ = 10.0 Hz, H-17), 2.40 (1*H*, *ddd*, *J*_23,23 (1)_ = 9.54, *J*_23,24eq_ = 3.4, *J*_23,24ax_ = 11.0 Hz, H-23), 1.57 (3*H*, *s*, CH_3_-21), 1.11 (3*H*, *d*, *J*_23 (2),23 (1)_ = 6.5 Hz, C-23^2^), 0.95 (3*H*, *s*, CH_3_-19), 0.76 (3*H*, *d*, *J*_27,25_ = 7.0 Hz, CH_3_-27), 0.67 (3*H*, *s*, CH_3_-18). ^13^C NMR (125 MHz, CDCl_3_): δ (ppm) 152.5 (C-22), 105.0 (C-20), 85.0 (C-16), 73.4 (C-23^1^), 73.3 (C-26), 67.1 (C-3), 64.3 (C-17), 55.1 (C-14), 44.0 (C-9), 41.5 (C-10), 41.3 (C-23), 40.1 (C-5), 39.8 (C-4), 36.5 (C-6), 36.3 (C-15), 35.3 (C-1), 35.0 (C-12), 30.9 (C-2), 30.0 (C-13), 27.9 (C-7), 26.7 (C-8), 26.5 (C-25), 23.9 (C-24), 21.7 (C-11), 20.1 (CH_3_-23^2^), 17.0 (CH_3_-19), 14.8 (CH_3_-27), 14.3 (CH_3_-18), 11.9 (CH_3_-21).

## Refinement

Crystal data, data collection and structure refinement details are summarized in Table 2 ▸. All H atoms were placed in calculated positions, with C—H = 1.00 (methine CH), 0.99 (methyl­ene CH_2_) or 0.98 Å (methyl CH_3_). Atom H3*A* (of the hy­droxy group) was refined with free coordinates. Isotropic displacement parameters for the H atoms were calculated as *U*_iso_(H) = *xU*_eq_(carrier atom), with *x* = 1.5 for methyl groups and the hydroxy H atom, and *x* = 1.2 for the other H atoms. The methyl groups were allowed to rotate but not to tip. Due to the absence of anomalous scatterers, the absolute configuration could not be determined and was set according to the starting material.

## Supplementary Material

Crystal structure: contains datablock(s) I, global. DOI: 10.1107/S2414314623003449/bt4138sup1.cif

Structure factors: contains datablock(s) I. DOI: 10.1107/S2414314623003449/bt4138Isup2.hkl

CCDC reference: 2256805

Additional supporting information:  crystallographic information; 3D view; checkCIF report

## Figures and Tables

**Table 1:** Hydrogen-bond geometry (Å, °)

*D*—H⋯*A*	*D*—H	H⋯*A*	*D*⋯*A*	*D*—H⋯*A*
O3—H3*A*⋯O26^i^	0.87 (4)	2.11 (4)	2.943 (4)	159 (5)

Symmetry code: (i) 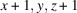 .

**Table 2:** Experimental details

Crystal data	
Chemical formula	C_29_H_46_O_3_
*M*_r_	442.66
Crystal system, space group	Monoclinic, *P*2_1_
Temperature (K)	153
*a*, *b*, *c* (Å)	6.3829 (7), 12.6897 (8), 15.9539 (17)
β (°)	101.308 (8)
*V* (Å^3^)	1267.1 (2)
*Z*	2
Radiation type	Ag *K*α, λ = 0.56083 Å
μ (mm^−1^)	0.05
Crystal size (mm)	0.24 × 0.16 × 0.07

Data collection	
Diffractometer	Stoe Stadivari
Absorption correction	Multi-scan (*X-AREA*; Stoe & Cie, 2018 ▸)
*T*_min_, *T*_max_	0.519, 1.000
No. of measured, independent and observed [*I* > 2σ(*I*)] reflections	15499, 4669, 2283
*R*_int_	0.102
(sin θ/λ)_max_ (Å^−1^)	0.624

Refinement	
*R*[*F* ^2^ > 2σ(*F* ^2^)], *wR*(*F* ^2^), *S*	0.043, 0.077, 0.70
No. of reflections	4669
No. of parameters	298
No. of restraints	1
H-atom treatment	H atoms treated by a mixture of independent and constrained refinement
Δρ_max_, Δρ_min_ (e Å^−3^)	0.16, −0.18

Computer programs: *X-AREA* (Stoe & Cie, 2018 ▸), *SHELXT2018* (Sheldrick, 2015*a* ▸), *SHELXL2018* (Sheldrick, 2015*b* ▸), *XP* in *SHELXTL-Plus* (Sheldrick, 2008 ▸), *Mercury* (Macrae *et al.*, 2020 ▸) and *publCIF* (Westrip, 2010 ▸).

## full crystallographic data

### Crystal data

**Table:**

C_29_H_46_O_3_	*F*(000) = 488
*M**_r_* = 442.66	*D*_x_ = 1.160 Mg m^−^^3^
Monoclinic, *P*2_1_	Ag *K*α radiation, λ = 0.56083 Å
*a* = 6.3829 (7) Å	Cell parameters from 7481 reflections
*b* = 12.6897 (8) Å	θ = 2.4–27.7°
*c* = 15.9539 (17) Å	µ = 0.05 mm^−^^1^
β = 101.308 (8)°	*T* = 153 K
*V* = 1267.1 (2) Å^3^	Irregular, colourless
*Z* = 2	0.24 × 0.16 × 0.07 mm

### Data collection

**Table:**

Stoe Stadivari diffractometer	4669 independent reflections
Radiation source: Sealed X-ray tube, Axo Astix-f Microfocus source	2283 reflections with *I* > 2σ(*I*)
Graded multilayer mirror monochromator	*R*_int_ = 0.102
Detector resolution: 5.81 pixels mm^-1^	θ_max_ = 20.5°, θ_min_ = 2.4°
ω scans	*h* = −7→7
Absorption correction: multi-scan (X-AREA; Stoe & Cie, 2018)	*k* = −15→15
*T*_min_ = 0.519, *T*_max_ = 1.000	*l* = −19→19
15499 measured reflections	

### Refinement

**Table:**

Refinement on *F*^2^	Secondary atom site location: difference Fourier map
Least-squares matrix: full	Hydrogen site location: mixed
*R*[*F*^2^ > 2σ(*F*^2^)] = 0.043	H atoms treated by a mixture of independent and constrained refinement
*wR*(*F*^2^) = 0.077	*w* = 1/[σ^2^(*F*_o_^2^) + (0.0188*P*)^2^] where *P* = (*F*_o_^2^ + 2*F*_c_^2^)/3
*S* = 0.70	(Δ/σ)_max_ < 0.001
4669 reflections	Δρ_max_ = 0.16 e Å^−^^3^
298 parameters	Δρ_min_ = −0.17 e Å^−^^3^
1 restraint	Extinction correction: (SHELXL2018; Sheldrick 2015*b*), Fc^*^=kFc[1+0.001xFc^2^λ^3^/sin(2θ)]^-1/4^
0 constraints	Extinction coefficient: 0.0100 (10)
Primary atom site location: dual	

### Fractional atomic coordinates and isotropic or equivalent isotropic displacement parameters (Å^2^)

**Table:**

	*x*	*y*	*z*	*U*_iso_*/*U*_eq_	
C1	0.7075 (7)	0.3112 (3)	0.7615 (3)	0.0303 (10)	
H1A	0.621334	0.309403	0.806762	0.036*	
H1B	0.674723	0.246376	0.726844	0.036*	
C2	0.9432 (7)	0.3097 (3)	0.8037 (3)	0.0335 (11)	
H2A	0.972593	0.247124	0.841295	0.040*	
H2B	1.030808	0.303861	0.759143	0.040*	
C3	1.0065 (6)	0.4089 (3)	0.8563 (2)	0.0353 (10)	
H3	1.165276	0.410355	0.875704	0.042*	
O3	0.9070 (5)	0.4036 (2)	0.93021 (18)	0.0447 (8)	
H3A	0.972 (8)	0.449 (3)	0.967 (3)	0.067*	
C4	0.9355 (6)	0.5075 (3)	0.8039 (2)	0.0313 (10)	
H4A	1.024977	0.515500	0.760204	0.038*	
H4B	0.961535	0.569582	0.842168	0.038*	
C5	0.7003 (7)	0.5073 (3)	0.7592 (2)	0.0304 (10)	
H5	0.612950	0.506869	0.804783	0.036*	
C6	0.6443 (7)	0.6089 (3)	0.7069 (3)	0.0369 (12)	
H6A	0.487038	0.614593	0.689524	0.044*	
H6B	0.695409	0.670465	0.743349	0.044*	
C7	0.7433 (7)	0.6119 (3)	0.6272 (2)	0.0332 (11)	
H7A	0.900717	0.615808	0.644573	0.040*	
H7B	0.693851	0.675862	0.593581	0.040*	
C8	0.6827 (6)	0.5142 (3)	0.5712 (2)	0.0257 (9)	
H8	0.524350	0.514261	0.550355	0.031*	
C9	0.7450 (6)	0.4127 (3)	0.6240 (2)	0.0234 (9)	
H9	0.902181	0.417847	0.646833	0.028*	
C10	0.6379 (6)	0.4079 (3)	0.7034 (2)	0.0255 (9)	
C11	0.7125 (7)	0.3130 (3)	0.5679 (3)	0.0287 (10)	
H11A	0.775839	0.252065	0.602689	0.034*	
H11B	0.557409	0.299583	0.549940	0.034*	
C12	0.8121 (7)	0.3202 (3)	0.4879 (2)	0.0283 (10)	
H12A	0.969689	0.322403	0.505309	0.034*	
H12B	0.773442	0.256685	0.452232	0.034*	
C13	0.7352 (6)	0.4182 (3)	0.4354 (2)	0.0229 (9)	
C14	0.7897 (6)	0.5144 (3)	0.4943 (2)	0.0235 (9)	
H14	0.947049	0.511511	0.517207	0.028*	
C15	0.7515 (7)	0.6089 (3)	0.4321 (2)	0.0314 (11)	
H15A	0.597790	0.626844	0.416707	0.038*	
H15B	0.832343	0.671696	0.457074	0.038*	
C16	0.8341 (7)	0.5686 (3)	0.3548 (2)	0.0288 (10)	
H16	0.975553	0.601243	0.352721	0.035*	
O16	0.6841 (4)	0.58617 (17)	0.27418 (16)	0.0291 (7)	
C17	0.8563 (6)	0.4468 (3)	0.3631 (3)	0.0251 (10)	
H17	1.010420	0.427022	0.380546	0.030*	
C18	0.4938 (5)	0.4103 (3)	0.3978 (2)	0.0277 (9)	
H18A	0.466708	0.349055	0.359841	0.042*	
H18B	0.446279	0.474441	0.365412	0.042*	
H18C	0.415055	0.402417	0.444388	0.042*	
C19	0.3914 (5)	0.4007 (4)	0.6764 (3)	0.0373 (10)	
H19A	0.339983	0.455995	0.634456	0.056*	
H19B	0.326656	0.410169	0.726717	0.056*	
H19C	0.351771	0.331534	0.650887	0.056*	
C20	0.7720 (5)	0.4121 (3)	0.2728 (2)	0.0246 (9)	
C21	0.7993 (7)	0.3020 (3)	0.2430 (3)	0.0340 (11)	
H21A	0.720725	0.253044	0.273020	0.051*	
H21B	0.951313	0.283437	0.255138	0.051*	
H21C	0.743733	0.297329	0.181338	0.051*	
C22	0.6775 (6)	0.4928 (3)	0.2281 (2)	0.0261 (10)	
C23	0.5667 (6)	0.5036 (3)	0.1365 (2)	0.0268 (9)	
H23	0.585841	0.436189	0.106392	0.032*	
C24	0.6676 (6)	0.5930 (3)	0.0927 (2)	0.0300 (10)	
H24A	0.662424	0.659391	0.124732	0.036*	
H24B	0.819359	0.576203	0.093234	0.036*	
C25	0.5498 (7)	0.6076 (3)	0.0008 (3)	0.0338 (10)	
H25	0.565143	0.541937	−0.032062	0.041*	
C26	0.3142 (7)	0.6251 (3)	0.0011 (3)	0.0381 (12)	
H26A	0.297184	0.690286	0.033231	0.046*	
H26B	0.235255	0.634484	−0.058357	0.046*	
O26	0.2251 (4)	0.53738 (19)	0.03962 (17)	0.0344 (8)	
C27	0.3263 (6)	0.5230 (3)	0.1284 (2)	0.0276 (10)	
H27	0.304525	0.587849	0.161257	0.033*	
C28	0.6418 (7)	0.6999 (3)	−0.0415 (3)	0.0475 (13)	
H28A	0.612316	0.765851	−0.014044	0.071*	
H28B	0.575716	0.702249	−0.102335	0.071*	
H28C	0.796571	0.690815	−0.035290	0.071*	
C29	0.2096 (6)	0.4323 (3)	0.1596 (3)	0.0335 (11)	
H29A	0.059098	0.451242	0.155687	0.050*	
H29B	0.274301	0.416708	0.219255	0.050*	
H29C	0.219447	0.370062	0.124274	0.050*	

### Atomic displacement parameters (Å^2^)

**Table:**

	*U*^11^	*U*^22^	*U*^33^	*U*^12^	*U*^13^	*U*^23^
C1	0.036 (3)	0.033 (2)	0.022 (2)	−0.002 (2)	0.008 (2)	0.004 (2)
C2	0.037 (3)	0.032 (2)	0.033 (3)	0.002 (2)	0.010 (2)	0.007 (2)
C3	0.031 (2)	0.048 (2)	0.027 (2)	−0.004 (2)	0.005 (2)	0.007 (2)
O3	0.050 (2)	0.0554 (19)	0.0292 (19)	−0.0094 (18)	0.0075 (16)	−0.0026 (17)
C4	0.033 (3)	0.035 (2)	0.024 (2)	−0.004 (2)	0.001 (2)	−0.002 (2)
C5	0.031 (3)	0.032 (2)	0.028 (2)	0.002 (2)	0.007 (2)	0.000 (2)
C6	0.042 (3)	0.032 (2)	0.035 (3)	0.008 (2)	0.004 (2)	−0.006 (2)
C7	0.045 (3)	0.0236 (19)	0.029 (3)	0.005 (2)	0.002 (2)	0.0007 (19)
C8	0.025 (2)	0.0226 (19)	0.026 (2)	0.000 (2)	−0.0029 (19)	−0.003 (2)
C9	0.022 (2)	0.0236 (17)	0.025 (2)	0.004 (2)	0.0046 (18)	0.001 (2)
C10	0.020 (2)	0.031 (2)	0.024 (2)	0.002 (2)	0.0025 (19)	0.001 (2)
C11	0.033 (3)	0.022 (2)	0.030 (2)	0.0007 (19)	0.004 (2)	0.002 (2)
C12	0.030 (3)	0.026 (2)	0.029 (3)	0.0032 (18)	0.007 (2)	0.0023 (19)
C13	0.023 (2)	0.0224 (18)	0.024 (2)	0.002 (2)	0.0064 (18)	0.0032 (19)
C14	0.022 (2)	0.0216 (18)	0.025 (2)	−0.0012 (19)	0.0010 (19)	0.004 (2)
C15	0.040 (3)	0.0238 (19)	0.029 (3)	−0.004 (2)	0.001 (2)	0.0005 (19)
C16	0.030 (3)	0.026 (2)	0.027 (2)	−0.0041 (18)	−0.003 (2)	0.0039 (19)
O16	0.0371 (18)	0.0245 (14)	0.0235 (16)	0.0008 (13)	0.0006 (14)	0.0011 (12)
C17	0.015 (2)	0.028 (2)	0.032 (3)	0.0028 (16)	0.004 (2)	0.0017 (18)
C18	0.028 (2)	0.0282 (18)	0.026 (2)	0.000 (2)	0.0025 (18)	−0.001 (2)
C19	0.025 (2)	0.049 (2)	0.039 (3)	0.001 (2)	0.009 (2)	−0.004 (2)
C20	0.022 (2)	0.0290 (19)	0.025 (2)	−0.001 (2)	0.0094 (18)	0.001 (2)
C21	0.042 (3)	0.031 (2)	0.031 (3)	0.004 (2)	0.010 (2)	0.003 (2)
C22	0.024 (2)	0.029 (2)	0.027 (2)	−0.0041 (19)	0.008 (2)	−0.0045 (19)
C23	0.028 (2)	0.0256 (19)	0.026 (2)	0.000 (2)	0.0030 (19)	0.000 (2)
C24	0.030 (3)	0.033 (2)	0.027 (2)	−0.0035 (19)	0.006 (2)	0.0073 (19)
C25	0.037 (3)	0.035 (2)	0.028 (2)	−0.005 (2)	0.003 (2)	0.006 (2)
C26	0.037 (3)	0.043 (2)	0.030 (3)	0.001 (2)	−0.005 (2)	0.009 (2)
O26	0.0336 (18)	0.0410 (17)	0.0252 (17)	−0.0081 (14)	−0.0027 (14)	0.0052 (13)
C27	0.025 (2)	0.034 (2)	0.022 (2)	0.0020 (19)	0.0002 (19)	−0.0017 (19)
C28	0.050 (4)	0.056 (3)	0.036 (3)	−0.006 (3)	0.008 (3)	0.014 (2)
C29	0.030 (3)	0.043 (3)	0.029 (2)	−0.002 (2)	0.010 (2)	0.003 (2)

### Geometric parameters (Å, º)

**Table:**

C1—C2	1.523 (5)	C15—H15A	0.9900
C1—C10	1.551 (5)	C15—H15B	0.9900
C1—H1A	0.9900	C16—O16	1.462 (4)
C1—H1B	0.9900	C16—C17	1.556 (4)
C2—C3	1.523 (5)	C16—H16	1.0000
C2—H2A	0.9900	O16—C22	1.391 (4)
C2—H2B	0.9900	C17—C20	1.501 (5)
C3—O3	1.446 (4)	C17—H17	1.0000
C3—C4	1.524 (5)	C18—H18A	0.9800
C3—H3	1.0000	C18—H18B	0.9800
O3—H3A	0.87 (4)	C18—H18C	0.9800
C4—C5	1.531 (5)	C19—H19A	0.9800
C4—H4A	0.9900	C19—H19B	0.9800
C4—H4B	0.9900	C19—H19C	0.9800
C5—C6	1.539 (5)	C20—C22	1.324 (5)
C5—C10	1.550 (5)	C20—C21	1.498 (5)
C5—H5	1.0000	C21—H21A	0.9800
C6—C7	1.527 (5)	C21—H21B	0.9800
C6—H6A	0.9900	C21—H21C	0.9800
C6—H6B	0.9900	C22—C23	1.500 (5)
C7—C8	1.532 (5)	C23—C27	1.535 (5)
C7—H7A	0.9900	C23—C24	1.538 (5)
C7—H7B	0.9900	C23—H23	1.0000
C8—C14	1.517 (5)	C24—C25	1.522 (5)
C8—C9	1.548 (5)	C24—H24A	0.9900
C8—H8	1.0000	C24—H24B	0.9900
C9—C11	1.540 (5)	C25—C26	1.521 (5)
C9—C10	1.554 (5)	C25—C28	1.525 (5)
C9—H9	1.0000	C25—H25	1.0000
C10—C19	1.550 (5)	C26—O26	1.441 (4)
C11—C12	1.537 (5)	C26—H26A	0.9900
C11—H11A	0.9900	C26—H26B	0.9900
C11—H11B	0.9900	O26—C27	1.449 (4)
C12—C13	1.525 (5)	C27—C29	1.507 (5)
C12—H12A	0.9900	C27—H27	1.0000
C12—H12B	0.9900	C28—H28A	0.9800
C13—C14	1.538 (5)	C28—H28B	0.9800
C13—C18	1.543 (5)	C28—H28C	0.9800
C13—C17	1.552 (5)	C29—H29A	0.9800
C14—C15	1.545 (5)	C29—H29B	0.9800
C14—H14	1.0000	C29—H29C	0.9800
C15—C16	1.521 (5)		
			
C2—C1—C10	114.6 (3)	C16—C15—H15A	111.2
C2—C1—H1A	108.6	C14—C15—H15A	111.2
C10—C1—H1A	108.6	C16—C15—H15B	111.2
C2—C1—H1B	108.6	C14—C15—H15B	111.2
C10—C1—H1B	108.6	H15A—C15—H15B	109.1
H1A—C1—H1B	107.6	O16—C16—C15	113.0 (3)
C3—C2—C1	111.4 (3)	O16—C16—C17	105.0 (3)
C3—C2—H2A	109.3	C15—C16—C17	107.8 (3)
C1—C2—H2A	109.3	O16—C16—H16	110.3
C3—C2—H2B	109.3	C15—C16—H16	110.3
C1—C2—H2B	109.3	C17—C16—H16	110.3
H2A—C2—H2B	108.0	C22—O16—C16	106.4 (3)
O3—C3—C2	107.4 (3)	C20—C17—C13	120.5 (3)
O3—C3—C4	110.7 (3)	C20—C17—C16	101.5 (3)
C2—C3—C4	111.0 (3)	C13—C17—C16	104.2 (3)
O3—C3—H3	109.2	C20—C17—H17	109.9
C2—C3—H3	109.2	C13—C17—H17	109.9
C4—C3—H3	109.2	C16—C17—H17	109.9
C3—O3—H3A	107 (3)	C13—C18—H18A	109.5
C3—C4—C5	114.5 (3)	C13—C18—H18B	109.5
C3—C4—H4A	108.6	H18A—C18—H18B	109.5
C5—C4—H4A	108.6	C13—C18—H18C	109.5
C3—C4—H4B	108.6	H18A—C18—H18C	109.5
C5—C4—H4B	108.6	H18B—C18—H18C	109.5
H4A—C4—H4B	107.6	C10—C19—H19A	109.5
C4—C5—C6	110.7 (3)	C10—C19—H19B	109.5
C4—C5—C10	112.8 (3)	H19A—C19—H19B	109.5
C6—C5—C10	111.4 (3)	C10—C19—H19C	109.5
C4—C5—H5	107.2	H19A—C19—H19C	109.5
C6—C5—H5	107.2	H19B—C19—H19C	109.5
C10—C5—H5	107.2	C22—C20—C21	128.3 (4)
C7—C6—C5	112.4 (3)	C22—C20—C17	109.3 (3)
C7—C6—H6A	109.1	C21—C20—C17	122.4 (3)
C5—C6—H6A	109.1	C20—C21—H21A	109.5
C7—C6—H6B	109.1	C20—C21—H21B	109.5
C5—C6—H6B	109.1	H21A—C21—H21B	109.5
H6A—C6—H6B	107.9	C20—C21—H21C	109.5
C6—C7—C8	111.5 (3)	H21A—C21—H21C	109.5
C6—C7—H7A	109.3	H21B—C21—H21C	109.5
C8—C7—H7A	109.3	C20—C22—O16	114.2 (3)
C6—C7—H7B	109.3	C20—C22—C23	132.4 (4)
C8—C7—H7B	109.3	O16—C22—C23	113.4 (3)
H7A—C7—H7B	108.0	C22—C23—C27	111.7 (3)
C14—C8—C7	111.6 (3)	C22—C23—C24	110.6 (3)
C14—C8—C9	109.5 (3)	C27—C23—C24	110.2 (3)
C7—C8—C9	110.3 (3)	C22—C23—H23	108.1
C14—C8—H8	108.5	C27—C23—H23	108.1
C7—C8—H8	108.5	C24—C23—H23	108.1
C9—C8—H8	108.5	C25—C24—C23	110.9 (3)
C11—C9—C8	112.1 (3)	C25—C24—H24A	109.5
C11—C9—C10	114.2 (3)	C23—C24—H24A	109.5
C8—C9—C10	111.6 (3)	C25—C24—H24B	109.5
C11—C9—H9	106.0	C23—C24—H24B	109.5
C8—C9—H9	106.0	H24A—C24—H24B	108.1
C10—C9—H9	106.0	C26—C25—C24	108.5 (3)
C19—C10—C5	109.9 (3)	C26—C25—C28	110.9 (3)
C19—C10—C1	106.0 (3)	C24—C25—C28	111.1 (3)
C5—C10—C1	106.8 (3)	C26—C25—H25	108.8
C19—C10—C9	111.2 (3)	C24—C25—H25	108.8
C5—C10—C9	109.4 (3)	C28—C25—H25	108.8
C1—C10—C9	113.5 (3)	O26—C26—C25	111.2 (3)
C12—C11—C9	113.7 (3)	O26—C26—H26A	109.4
C12—C11—H11A	108.8	C25—C26—H26A	109.4
C9—C11—H11A	108.8	O26—C26—H26B	109.4
C12—C11—H11B	108.8	C25—C26—H26B	109.4
C9—C11—H11B	108.8	H26A—C26—H26B	108.0
H11A—C11—H11B	107.7	C26—O26—C27	112.1 (3)
C13—C12—C11	111.3 (3)	O26—C27—C29	105.4 (3)
C13—C12—H12A	109.4	O26—C27—C23	110.4 (3)
C11—C12—H12A	109.4	C29—C27—C23	113.6 (3)
C13—C12—H12B	109.4	O26—C27—H27	109.1
C11—C12—H12B	109.4	C29—C27—H27	109.1
H12A—C12—H12B	108.0	C23—C27—H27	109.1
C12—C13—C14	107.6 (3)	C25—C28—H28A	109.5
C12—C13—C18	110.5 (3)	C25—C28—H28B	109.5
C14—C13—C18	112.2 (3)	H28A—C28—H28B	109.5
C12—C13—C17	116.4 (3)	C25—C28—H28C	109.5
C14—C13—C17	100.5 (3)	H28A—C28—H28C	109.5
C18—C13—C17	109.3 (3)	H28B—C28—H28C	109.5
C8—C14—C13	114.1 (3)	C27—C29—H29A	109.5
C8—C14—C15	118.8 (3)	C27—C29—H29B	109.5
C13—C14—C15	103.6 (3)	H29A—C29—H29B	109.5
C8—C14—H14	106.5	C27—C29—H29C	109.5
C13—C14—H14	106.5	H29A—C29—H29C	109.5
C15—C14—H14	106.5	H29B—C29—H29C	109.5
C16—C15—C14	102.9 (3)		
			
C10—C1—C2—C3	56.8 (4)	C18—C13—C14—C15	−70.0 (4)
C1—C2—C3—O3	70.1 (4)	C17—C13—C14—C15	46.1 (4)
C1—C2—C3—C4	−51.1 (4)	C8—C14—C15—C16	−166.3 (3)
O3—C3—C4—C5	−68.4 (4)	C13—C14—C15—C16	−38.5 (4)
C2—C3—C4—C5	50.8 (4)	C14—C15—C16—O16	131.1 (3)
C3—C4—C5—C6	−179.1 (3)	C14—C15—C16—C17	15.5 (4)
C3—C4—C5—C10	−53.6 (5)	C15—C16—O16—C22	−135.1 (3)
C4—C5—C6—C7	71.5 (4)	C17—C16—O16—C22	−17.8 (4)
C10—C5—C6—C7	−54.9 (5)	C12—C13—C17—C20	95.8 (4)
C5—C6—C7—C8	54.7 (4)	C14—C13—C17—C20	−148.4 (3)
C6—C7—C8—C14	−177.2 (3)	C18—C13—C17—C20	−30.2 (5)
C6—C7—C8—C9	−55.3 (4)	C12—C13—C17—C16	−151.3 (3)
C14—C8—C9—C11	−49.8 (4)	C14—C13—C17—C16	−35.5 (4)
C7—C8—C9—C11	−173.0 (4)	C18—C13—C17—C16	82.6 (4)
C14—C8—C9—C10	−179.5 (3)	O16—C16—C17—C20	17.8 (4)
C7—C8—C9—C10	57.4 (4)	C15—C16—C17—C20	138.5 (3)
C4—C5—C10—C19	167.6 (3)	O16—C16—C17—C13	−108.1 (4)
C6—C5—C10—C19	−67.1 (4)	C15—C16—C17—C13	12.7 (4)
C4—C5—C10—C1	53.1 (4)	C13—C17—C20—C22	102.3 (4)
C6—C5—C10—C1	178.3 (4)	C16—C17—C20—C22	−12.0 (4)
C4—C5—C10—C9	−70.1 (4)	C13—C17—C20—C21	−78.2 (5)
C6—C5—C10—C9	55.1 (4)	C16—C17—C20—C21	167.5 (3)
C2—C1—C10—C19	−173.3 (3)	C21—C20—C22—O16	−178.3 (3)
C2—C1—C10—C5	−56.1 (4)	C17—C20—C22—O16	1.2 (5)
C2—C1—C10—C9	64.5 (4)	C21—C20—C22—C23	0.7 (7)
C11—C9—C10—C19	−64.1 (4)	C17—C20—C22—C23	−179.9 (4)
C8—C9—C10—C19	64.5 (4)	C16—O16—C22—C20	11.1 (4)
C11—C9—C10—C5	174.4 (3)	C16—O16—C22—C23	−168.1 (3)
C8—C9—C10—C5	−57.0 (4)	C20—C22—C23—C27	112.5 (5)
C11—C9—C10—C1	55.3 (4)	O16—C22—C23—C27	−68.5 (4)
C8—C9—C10—C1	−176.2 (3)	C20—C22—C23—C24	−124.5 (5)
C8—C9—C11—C12	49.6 (4)	O16—C22—C23—C24	54.5 (4)
C10—C9—C11—C12	177.9 (3)	C22—C23—C24—C25	−177.5 (3)
C9—C11—C12—C13	−53.6 (4)	C27—C23—C24—C25	−53.6 (4)
C11—C12—C13—C14	56.6 (4)	C23—C24—C25—C26	54.9 (4)
C11—C12—C13—C18	−66.2 (4)	C23—C24—C25—C28	177.0 (3)
C11—C12—C13—C17	168.4 (3)	C24—C25—C26—O26	−58.8 (4)
C7—C8—C14—C13	180.0 (3)	C28—C25—C26—O26	179.0 (3)
C9—C8—C14—C13	57.6 (4)	C25—C26—O26—C27	62.2 (4)
C7—C8—C14—C15	−57.3 (4)	C26—O26—C27—C29	177.6 (3)
C9—C8—C14—C15	−179.7 (3)	C26—O26—C27—C23	−59.3 (4)
C12—C13—C14—C8	−61.0 (4)	C22—C23—C27—O26	177.7 (3)
C18—C13—C14—C8	60.7 (4)	C24—C23—C27—O26	54.4 (4)
C17—C13—C14—C8	176.8 (3)	C22—C23—C27—C29	−64.2 (4)
C12—C13—C14—C15	168.3 (3)	C24—C23—C27—C29	172.6 (3)

### Hydrogen-bond geometry (Å, º)

**Table:**

*D*—H···*A*	*D*—H	H···*A*	*D*···*A*	*D*—H···*A*
O3—H3*A*···O26^i^	0.87 (4)	2.11 (4)	2.943 (4)	159 (5)

Symmetry code: (i) *x*+1, *y*, *z*+1.
